# Alternative Splicing of *sept9a* and *sept9b* in Zebrafish Produces Multiple mRNA Transcripts Expressed Throughout Development

**DOI:** 10.1371/journal.pone.0010712

**Published:** 2010-05-19

**Authors:** Megan L. Landsverk, Douglas C. Weiser, Mark C. Hannibal, David Kimelman

**Affiliations:** 1 Department of Pediatrics, University of Washington, Seattle, Washington, United States of America; 2 Department of Biochemistry, University of Washington, Seattle, Washington, United States of America; 3 Department of Molecular and Human Genetics, Baylor College of Medicine, Houston, Texas, United States of America; 4 Department of Biological Sciences, University of the Pacific, Stockton, California, United States of America; Texas A&M University, United States of America

## Abstract

**Background:**

Septins are involved in a number of cellular processes including cytokinesis and organization of the cytoskeleton. Alterations in human septin-9 (*SEPT9*) levels have been linked to multiple cancers, whereas mutations in *SEPT9* cause the episodic neuropathy, hereditary neuralgic amyotrophy (HNA). Despite its important function in human health, the *in vivo* role of *SEPT9* is unknown.

**Methodology/Principal Findings:**

Here we utilize zebrafish to study the role of *SEPT9* in early development. We show that zebrafish possess two genes, *sept9a* and *sept9b* that, like humans, express multiple transcripts. Knockdown or overexpression of *sept9a* transcripts results in specific developmental alterations including circulation defects and aberrant epidermal development.

**Conclusions/Significance:**

Our work demonstrates that *sept9* plays an important role in zebrafish development, and establishes zebrafish as a valuable model organism for the study of *SEPT9*.

## Introduction

Septin-9 (SEPT9, MSF) is a member of the septin gene family, a conserved family of filament forming GTPases. To date, at least 14 different septin genes have been identified in humans which, in addition to cytokinesis, also play roles in vesicle trafficking, microtubule and actin function, exocytosis, establishment of cell polarity and cell motility [1], [2]. All vertebrate septins have a highly conserved polybasic domain (PBD) followed by a GTP binding domain (GBD) homologous to those of the ras-related small GTPase family of proteins. Outside of the PBD and GBD, members of the septin family vary greatly in the length and make up of both the N- and C-termini. SEPT9 is one of three septin proteins possessing an extended N-terminus containing a proline-rich region. However, the function of this region is unknown.

The human *SEPT9* gene is complex, producing at least seven mRNA transcripts encoding six distinct polypeptides through alternative splicing [3]. *SEPT9* was initially identified as a fusion partner of the mixed-lineage leukemia (MLL) gene in acute myeloid leukemia patients [4]. Altered expression of *SEPT9* has also been implicated in the pathogenesis of a number of cancers, with evidence for both genetic gain and allelic loss [5], [6], [7], [8]. Point mutations and intragenic duplications in *SEPT9* have also been linked to hereditary neuralgic amyotrophy (HNA), an autosomal dominant episodic neuropathy primarily affecting the brachial plexus [9], [10].

In cultured cells, inhibition of *SEPT9* isoforms through antibody microinjection or siRNA transfection results in cytokinesis defects, including binucleated cells, abnormal daughter cells, cells remaining attached through a short midbody bridge, and cells containing condensed DNA suggestive of apoptosis [11], [12]. Overexpression of *SEPT9* isoforms in cell culture also leads to an increase in binucleated cells, an accumulation of cells in G2/M phase and an increase in the percentage of aneuploid cells leading to suppression of cell growth [13], [14]. However, overexpression of *SEPT9* isoforms has also been shown to increase cell motility, and alter cellular polarity and morphology [14], [15].

No mouse knockout has been described, and so the *in vivo* role of *SEPT9* remains unknown. Moreover, the transcriptional complexity of the *SEPT9* locus will make it very difficult to study the function of specific isoforms in the mouse. In this study we use the zebrafish system to investigate the *in vivo* role of specific *SEPT9* isoforms in early development. Zebrafish provide an excellent model for the study of genes with multiple transcripts since animals grow quickly, and can be easily genetically manipulated through the use of transgenic overexpression constructs and specific transcript inhibition using morpholino oliogonucleotides (MOs). Zebrafish possess two *SEPT9* gene orthologues found on two different chromosomes, *sept9a* and *sept9b*. We found that, similar to humans, these genes express multiple mRNA transcripts that are expressed throughout development in a variety of tissues. Inhibition of all Sept9a isoforms or just the largest Sept9a isoform, sept9a_tv1, led to multiple defects in embryonic development demonstrating an essential embryonic role for this isoform. In particular, we observed an increase in apoptosis in the epidermis of all morphants and alterations in blood circulation. Overexpression of sept9a_tv1 led to similar developmental defects. Our results demonstrate the importance of *sept9* during embryonic development.

## Materials and Methods

### Zebrafish embryos and ethics

Zebrafish were maintained, staged and injected according to standard procedures [16]. All experiments were approved by and conducted in accordance with the guidelines established by the Institutional Animal Care and Use Committee at the University of Washington, IACUC approval number: 2387-02.

### Identification and cloning of *sept9* isoforms

BLAST searches using human SEPT9 were used to identify zebrafish *sept9* transcripts. PCR primers were used to amplify *sept9a* isoforms from 24 hpf embryos. Primer sequences are available upon request.

### RNA isolation and RT-PCR

RNA was isolated using the RNeasy kit (Qiagen). cDNA was prepared using Superscript polymerase (Invitrogen) using 1 ug RNA. *sept9* isoforms and *ef1α* were amplified using transcript specific primers.

### Whole-mount in situ hybridization

Embryos were processed as described [16]. The *sept9a_tv1* coding region was used to generate digoxigenin-labeled probes (Roche).

### Morpholino and mRNA injections

Morpholinos targeted to the splice acceptor sites of *sept9a_tv1* exons 2 and 5 and *sept9a_tv1* mRNA were injected into zebrafish embryos. The sequences of the morpholinos are: MO2 (5′-TGCGATGCCTGTCAGCACAGAAGAC-3′), MO5 (5′-CTCTGACCTGCACACATGAAGAACA-3′), MO2 mismatch (5′-TCCGATCCCTGTGAGCACACAACAC-3′), MO5 mismatch (5′-CTGTGAGCTGCAAACATCAACAACA-3′). Full-length *sept9a_tv1* was subcloned into the pXLT vector for *in vitro* transcription. Messenger mRNA was synthesized using the mMessage Machine Kit (Ambion).

### Acridine orange (AO) staining

For AO staining, embryos were processed as described [17].

## Results

### Characterization of zebrafish *sept9* genes

**Figure 1:**
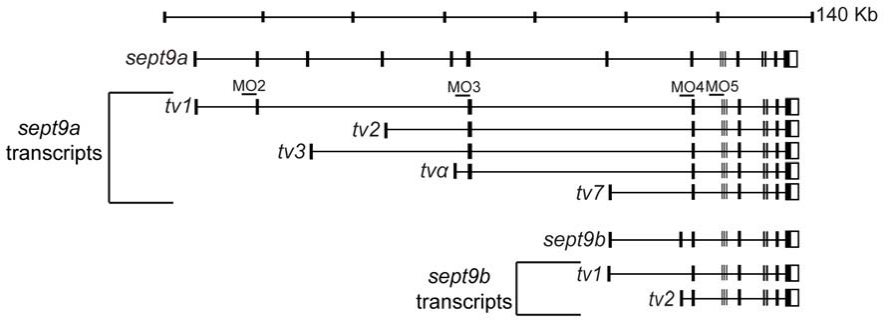
SEPT9 transcripts are conserved between zebrafish and mammals. Zebrafish possess two *sept9* genes, *sept9a* and *sept9b*, that encode multiple mRNA transcripts homologous to mammalian transcripts. Zebrafish also express a transcript not currently found in mammals, *sept9a_tvα*. Sites of morpholino splice blockers are noted.

**Figure 2:**
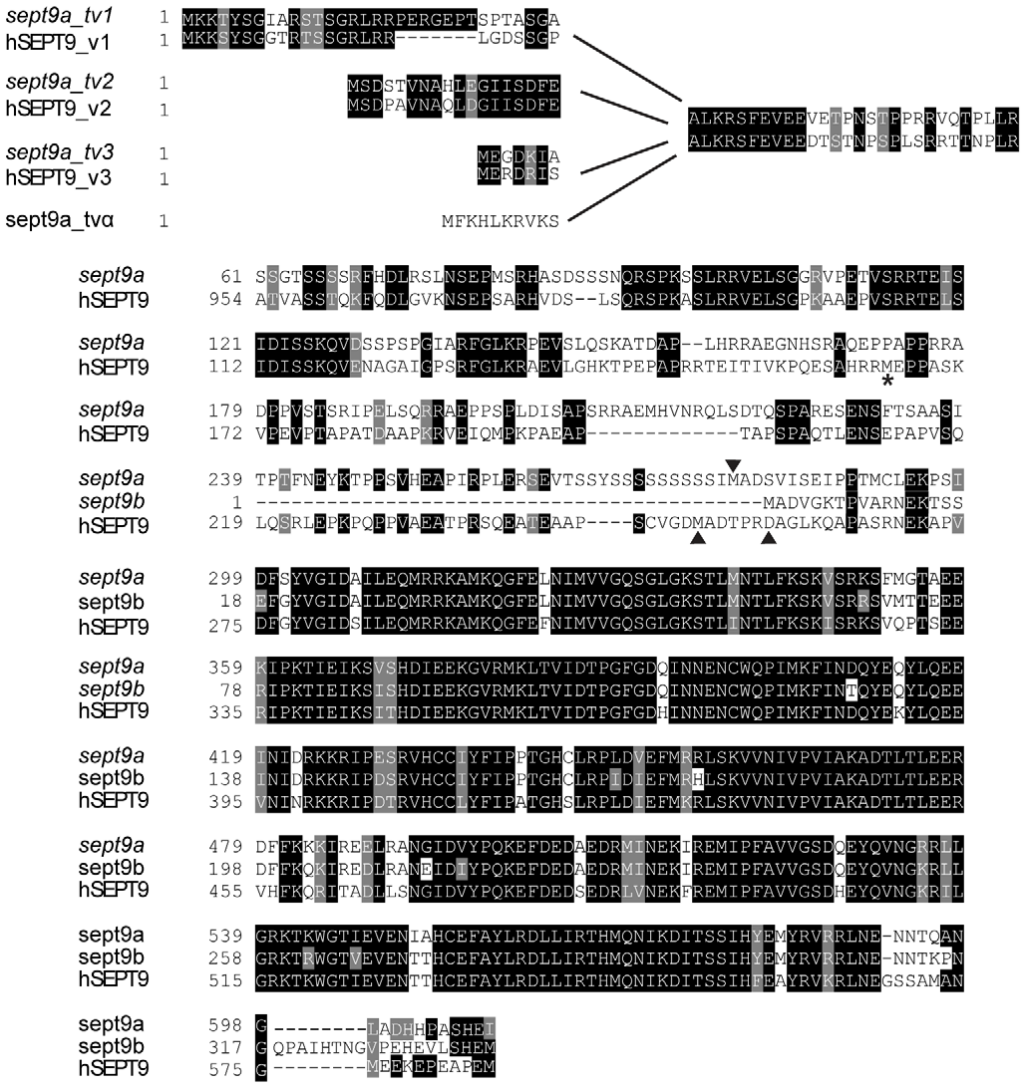
SEPT9 amino acid sequence is conserved between zebrafish and mammals. Amino-acid alignments of zebrafish Sept9a and Sept9b putative protein products show a high degree of conservation. An asterisk notes the starting methionine of human SEPT9_i5/6, which is not conserved in zebrafish. Arrowheads mark the starting methionines for human Sept9_v7, and zebrafish isoforms Sept9a_tv7 and Sept9b (both transcripts).

Through a combination of genetic sequence analysis and BLAST searches using known human *SEPT9* transcripts, we identified multiple mRNA transcripts produced from two different zebrafish *sept9* genes, *sept9a* and *sept9b*. *sept9a* is located on chromosome 3, whereas *sept9b* is found on chromosome 6. *sept9a* produces transcripts homologous to the longest human *SEPT9* isoforms 1, 2, and 3, the shortest human variant *SEPT9_v*7 (NCBI NM_001113496; named *SEPT9*_*v*5 in earlier literature [7]) and a unique transcript not identified in other vertebrates which we have denoted *sept9a_tvα*. Similar to humans, through the use of alternate 5′ exons, *sept9a _tv1, 2, 3* and α, generate predicted protein products with unique N-termini of 32, 18, 7, and 10 amino acids respectively (Fig. 1, and Fig. 2). *sept9a_tv7* encodes a truncated version of the longer transcripts. *sept9b* appears to express two human *SEPT9_v*7 homologues with alternate 5′ UTRs, *sept9b_tv1* and *sept9b_tv2* (Fig. 1). The predicted protein sequences of zebrafish *sept9a_tv*1, 2, and 3 are 73–74% similar and 61–62% identical to their human homologues, respectively (Fig. 2). *sept9a_tv7* and *sept9b_tv1* and *_tv2* primarily encode the GTP binding domain found in all transcripts, and are highly conserved. These transcripts are 92% similar and 87% identical to each other at the amino acid level and 87–81% similar, 78–80% identical to the human sequence. We did not identify transcripts homologous to human *SEPT9* transcript variants 4, 5, and 6 (NCBI NM_001113495, NM_001113492, and NM_001113494; *SEPT9_v5* and *v6* known as v4* and v4 in previous literature [7], [15], [18]). However, the start codon in human *SEPT9_*v5 and v6 is not conserved from mammals to zebrafish (Fig. 2) and to date, only a single EST of human *SEPT9_v4* has been identified in a teratocarcinoma cell line, suggesting that these transcripts may not be expressed in zebrafish. It is possible that further *sept9* transcripts are expressed in zebrafish yet were not identified in this analysis.

### Developmental expression of zebrafish *sept9* genes

**Figure 3:**
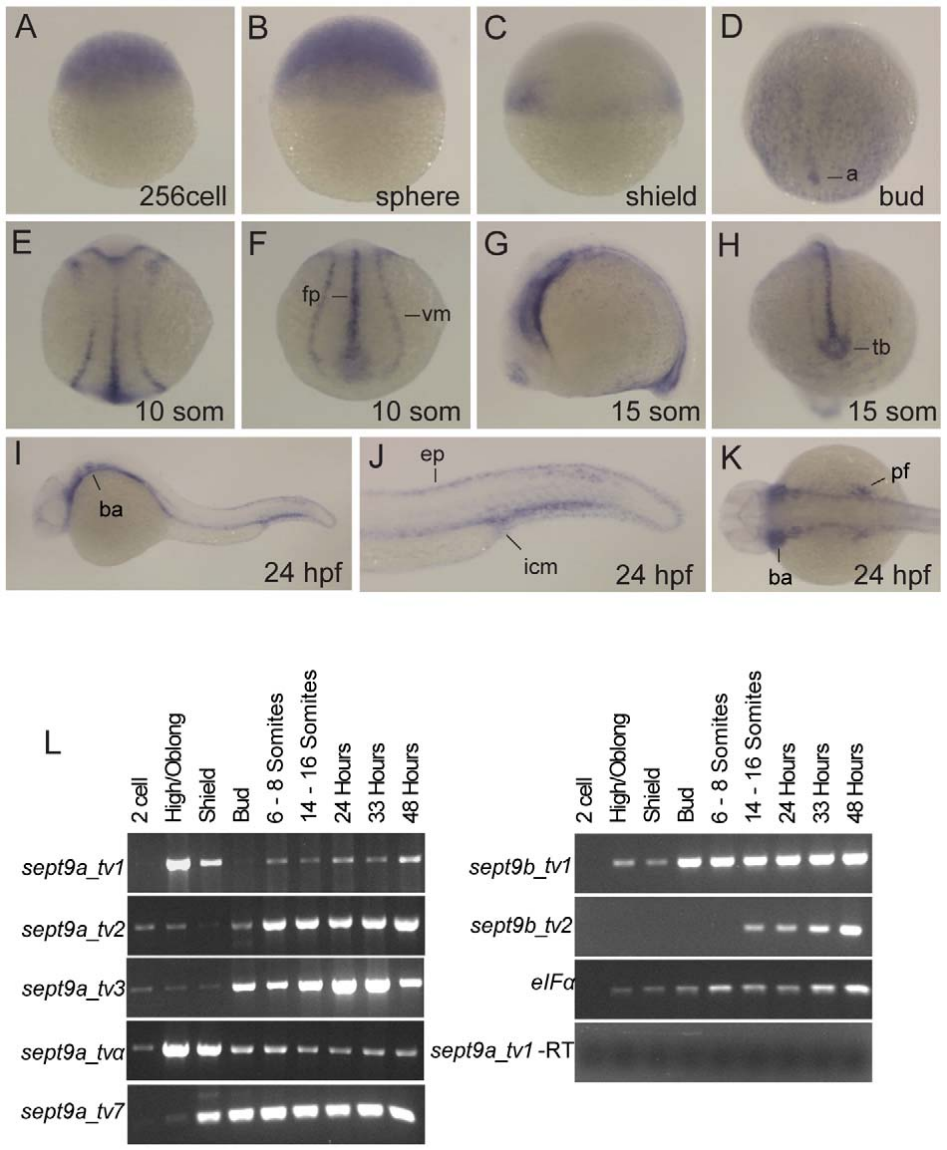
Expression of *sept9* genes during zebrafish development. Detection of *sept9* mRNA was carried out by whole-mount in situ hybridization using a probe targeted to all *sept9* isoforms on staged embryos from 256 cells to 24 hpf. Images in A–C are lateral views, animal pole to top; D and E are dorsal views, anterior to top; F and H are dorsal posterior views; G, I and J are lateral views, K is a dorsal view, anterior to left. **A–C**: *sept9* transcripts are ubiquitously expressed at early developmental states. **D**: At bud stage, *sept9* is expressed in endoderm and axis. **E–H**: *sept9* is expressed in the floor plate, ventral mesoderm and tail bud during segmentation. **I–K**: At 24 hpf, *sept9* is expressed throughout the epidermis, branchial arches, pectoral fin, and in the intermediate cell mass. **L**: Transcript specific primers were used to detect *sept9a* and *sept9b* transcripts in various stages of development by RT-PCR. *sept9a_tv 2, 3*, and *α* are expressed maternally. Amplification of *eIFα* and total RNA without addition of reverse transcriptase were used as controls. a, axis; ep, epidermis; fp, floorplate; icm, intermediate cell mass; tb, tail bud; vm, ventral mesoderm.

To examine where *sept9* message is expressed in the developing zebrafish embryo, we performed whole mount *in situ* hybridization using a probe to *sept9a_tv1*. This probe is expected to recognize all *sept9a* transcripts, and possibly those of *sept9b*. Probes designed to the individual *sept9a* transcripts were not synthesized because the unique regions of the longer *sept9a* isoforms are not large enough to probe individually. Early maternal expression of *sept9* was ubiquitous (Fig. 3A) and remained so through blastula stages (Fig. 3B). During gastrulation, *sept9* became more restricted to the axial mesoderm and endoderm (Fig. 3C, D). *sept9* was expressed primarily in the floor plate and ventral mesoderm during segmentation (Fig. 3E–H). At 24 hours post fertilization, *sept9* was expressed in the intermediate cell mass, epidermis, branchial arches, and pectoral fin bud (Fig. 3I–K).

While we were unable to analyze the spatial expression of specific *sept9* isoforms we could, using RT-PCR, determine the temporal expression pattern of the various *sept9* transcripts. Zebrafish embryos at different developmental stages were collected for cDNA preparation and subjected to RT-PCR using transcript specific primers (Fig. 3L). *sept9a_tv*2, 3, and α are expressed at the two cell stage consistent with the maternal *sept9a* expression observed in the *in situs*. Expression of *sept9a_tv1, 7* and *α, and sept9b_tv1* commence at high stage, consistent with zygotic expression, which begins at this time. All five transcripts of *sept9a* and both *sept9b* transcripts stabilize expression though the segmentation and pharyngula stages. The longest transcript, *sept9a*_*tv*1, has two phases of expression; one during the late blastula and early gastrula stages, and a second beginning during early somitogenesis.

### Effect of *sept9a* inhibition on zebrafish development

**Figure 4:**
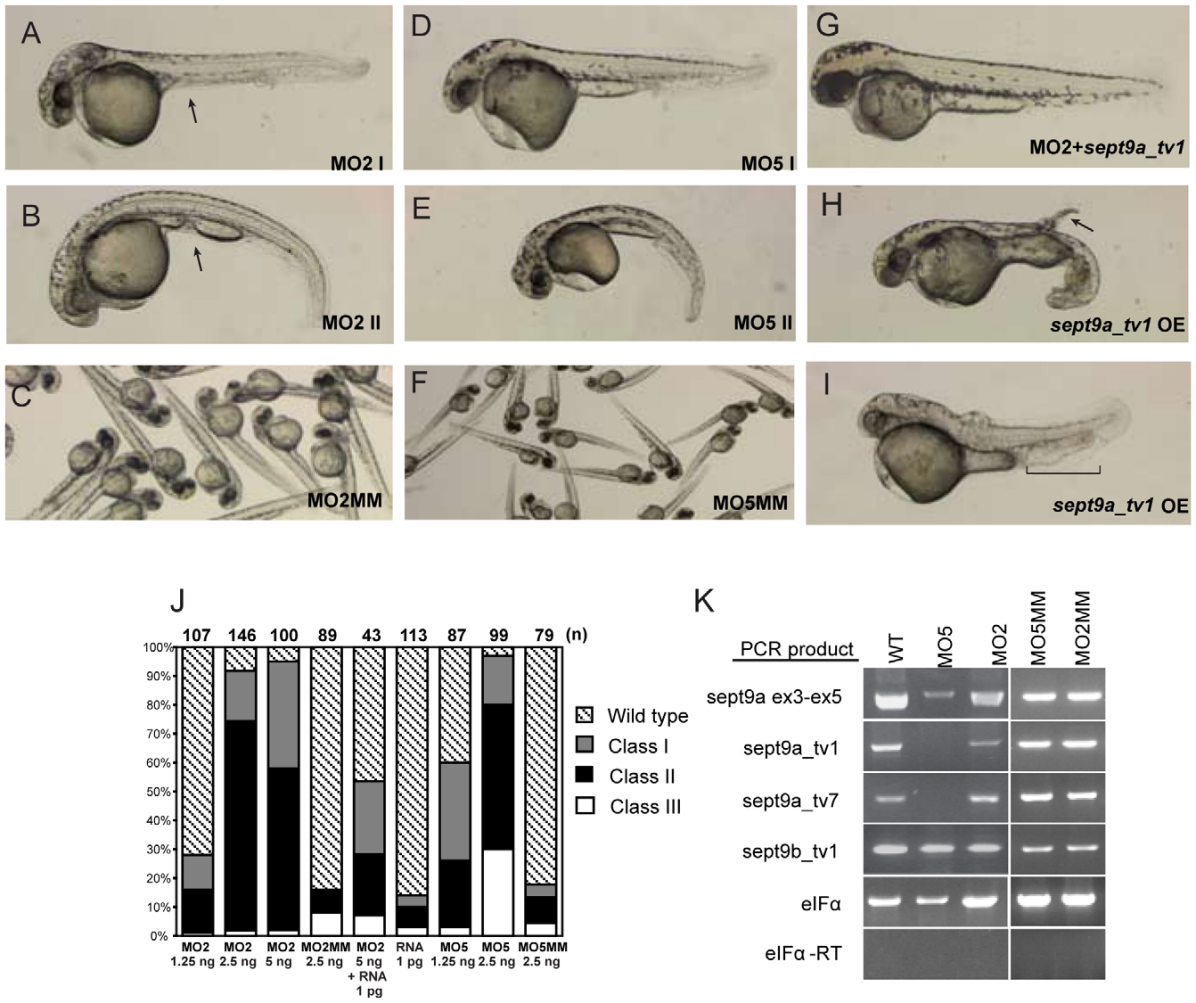
Charaterization of *sept9a* morphant and overexpression embryos. Embryos were injected at the one-cell stage with morpholinos targeted to all *sept9a* transcripts (MO5), *sept9a_tv1* only (MO2), mismatch controls (MO5MM, MO2MM), or *sept9a_tv1* mRNA with and without MO2. Morphants shown were injected with 2.5 ng morpholino. At 48 hpf, the phenotypes were assessed by morphological criteria, according to severity. **A,D**: Class I morphants had defects in epidermal integrity and yolk extension and minor curvature of the tail. **B,E**: Class II morphants had a curved body axis in addition to the defects observed in class I. Class III morphants had a severely shortened body axis (data not shown). Arrows indicate yolk extension defects. All classes exhibited defects in blood circulation. **G**: Coinjection of 1 pg of *sept9a_tv1* mRNA with 5 ng of MO2 partially rescued the observed phenotypes. **H,I**: Embryos injected with as little as 4 pg of *sept9a_tv1* mRNA often had phenotypes similar to those of *sept9a* morphants including epidermal aggregates (arrow), blood pooling, and tail edema (bracket). (OE) indicates over expression. **C,F**: Control mismatch morpholinos did not present a phenotype. **J**: Graphical representation of MO classes at various concentrations. The number of embryos tested in each experiments is indicated by (n) on top of each column. **K**: *sept9a* splice morpholinos inhibit *sept9a* transcript splicing. RT-PCR analysis was performed on 24 hpf wild-type embryos, embryos injected with 2.5 ng MO5 or MO2 (pooled classes I and II), or 5 bp mismatch controls. MO5 embryos show a complete loss of *sept9a_tv1* and *sept9a_tv7*. The presence of a low level of *sept9a* exons 3–5 transcripts in MO5-injected embryos may be due to maternal mRNA. MO2 embryos show a decrease in *sept9a_tv1* compared to wild-type while the other transcripts are not affected.

Because a majority of *sept9* transcripts, including the longest *sept9a_tv1*, derived from the *sept9a* locus, we decided to focus further studies on this set of isoforms. Therefore, we targeted all *sept9a* transcripts, or *sept9a_tv1* only, for depletion using antisense morpholinos directed to the splice acceptor site of the fifth (MO5) or second (MO2) exons of *sept9a_tv1*, respectively (Fig. 1). Embryos were injected at the 1-cell stage with 1.25, 2.5 or 5 ng of morpholino. The observed phenotypes were dosage dependent and were divided into three categories which we termed class I, class II and class III based on morphology (Fig. 4J). At 48 hpf, embryos that looked similar to wild-type but had epidermal defects and minor tail perturbations were categorized as class I, those that had a curved or shortened body axis/tail in addition to the defects in class I were categorized as class II and those that had a severely shortened tail/body axis were classified as class III. Both MO5 and MO2 produced classes I and II (Fig. 4A, B, D, E). However, the severe class III phenotype (data not shown) was not observed in MO2 embryos, even at 5 ng of morpholino.

Circulating blood cells could be observed in class I embryos, however, cells were often observed pooling in the intermediate cell mass (ICM) and tail region. Classes II and III showed an absence of blood circulation, and often lacked the presence of mature hemoglobinized erythrocytes. All classes exhibited yolk extension as well as epidermal defects, most commonly seen in the tail and yolk regions. Epidermal aggregates and edema were frequently noted, often at the tip of the tail and in the ICM. Cardiac edema was regularly observed in embryos from all classes and worsened as development proceeded; class II and III embryos did not survive past 7 days.

The phenotypes resulting from inhibition of *septa_tv1* or all *sept9a* transcripts were not regularly observed in embryos injected with mismatch controls to either morpholino (MO2MM and MO5MM; Fig. 4C, F, J). To determine if *sept9a* transcript levels were altered, RT-PCR was performed on 24 hpf MO5 and MO2 embryos injected with 2.5 ng morpholino (Fig. 4K). Levels of *sept9a_tv*1 and *tv*7 were undetectable in MO5 embryos, and *sept9a_tv*1 but not *_tv7* was decreased with MO2 when compared to wild-type. MO2 also did not affect an amplicon from exons 3–5, whereas MO5 greatly reduced the level of this product. Since these exons are shared with *sept9a_tv*2, 3, and α (Fig. 1), the residual product may be due to perduring maternal transcripts. Morpholinos designed to *sept9a_tv1* exon 3 (inhibiting transcript variants 1–3 and α) and exon 4 (inhibiting all transcripts) acceptor splice sites (Fig. 1) also produced the same classes of morphants observed in MO5 embryos (data not shown).

Co-injection of a low concentration (1 pg) of *sept9a_tv1* mRNA with MO2 showed a partial rescue of the morphant phenotypes providing further evidence that at least classes I and II are a result of *sept9a* transcript inhibition (Fig. 4G). The phenotype of MO5 injected embryos could not be rescued by co-injection of *sept9a_tv1* mRNA (data not shown). It is possible that *sept9a* transcripts have overlapping functions, and that over-expression of only *sept9a_tv1* cannot compensate for the loss of multiple transcripts. This also complicates interpretation of the class III phenotype, as it difficult to distinguish between a phenotype caused by morpholino artifact and one caused by knocking-down additional *sept9a* isoforms that can not be rescued with *sept9a_tv1*. However, the observation that four different *sept9a* MOs cause similar defects whereas mismatch morpholinos result in no defect and that the morphant phenotype is rescued by co-injection of *sept9a_tv1* mRNA, demonstrates that the observed morphant phenotypes are due to specific loss-of-function of *sept9* and not toxicity.

### Effect of *sept9a_tv1* overexpression on zebrafish development

Recent studies in cultured cells have shown that human *SEPT9* appears to be highly regulated [12], [13], [14]. While attempting rescue of *sept9a* morphant embryos, we found that increased levels of *sept9a_tv1 m*RNA led to a number of embryonic developmental defects including alterations in convergence and extension, dorsalization, and cyclopia. However, the phenotypes did not clearly group into classes like the *sept9a* morphant embryos. Interestingly, many of the phenotypes were similar to those observed in *sept9a* morphants including cardiac and tail fin edema, a curved tail and/or body axis, a loss of circulating blood cells with concentrated pools of cells in the tail region and ICM, and epidermal defects including regions of aggregated cells (Fig. 4H, I). Thus, some phenotypes were observed with both gain and loss of *sept9a_tv1* function, whereas other phenotypes were only found in gain-of-function experiments.

### Knock-down and overexpression of *sept9a* cause an increase in cell death

**Figure 5:**
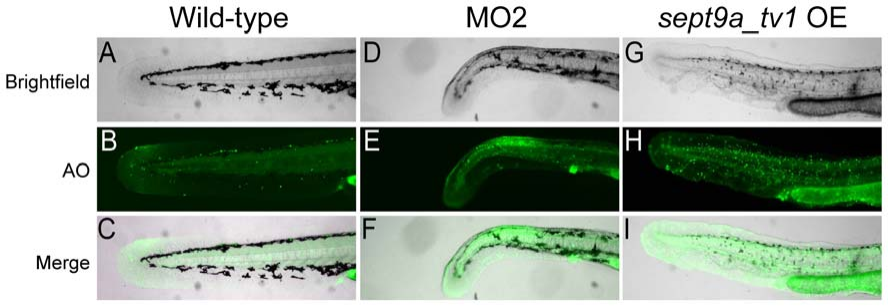
Knockdown and overexpression (OE) of *sept9a_tv1* results in an increase in apoptotic cells in the tail. Embryos at the one-cell stage were injected with 2.5 ng of MO2 or 4 pg of *sept9a_tv1* mRNA and analyzed for acridine orange (AO) staining at 24 hpf. **A–C**: The tail fin of wild-type embryos is negative for AO indicating few apoptotic cells. **D–I**: Both class II MO2 and *sept9a_v1* OE embryos show an increase in AO staining indicating increased cell death.

Alterations in human *SEPT9* have been shown to cause defects in cytokinesis, leading to changes in cell morphology and decreases in cellular growth [12], [14]. To determine if the defects observed in the tails of *sept9a_tv1* morphant and overexpression (OE) embryos included an increase in apoptotic cells, we used acridine orange (AO) to mark cell death. AO-positive cells were rarely observed in wild-type embryos, yet both *sept9a* MO2 and *sept9a_tv1* OE embryos showed an increase in apoptotic cells in the tail indicating cell death (Fig. 5). These data suggest that both loss- and gain-of-function of *sept9a* in zebrafish lead to an increase in cell death possibly through defects in cell division.

## Discussion

In this study we have shown that, like humans, zebrafish express multiple *sept9* transcripts. These transcripts are expressed throughout development in different tissues types including the ventral mesoderm and axis at early developmental stages, and the epidermis at later stages. We have demonstrated that inhibition and overexpression of *sept9a* transcripts in zebrafish embryos lead to a myriad of phenotypes including edema, loss of blood circulation, tail fin malformations, loss of epidermal integrity and increased cell death. Additionally, we have provided evidence that multiple *sept9a* MOs targeted to different splice sites yield similar phenotypes, and that overexpression of *sept9a_tv1* causes developmental defects similar to those observed with the MOs. Thus, too much or too little *sept9a* function is deleterious for many embryonic cells, indicating that cells need to carefully regulate *sept9a* levels. The correct levels of *sept9a*, therefore, are needed to maintain tissue integrity and to allow normal cell division.

The fact that zebrafish posses two *sept9* orthologues (*sept9a* and *sept9b*) is not unusual, given the proposed genomic duplication event that occurred in teleost fish [19]. The two orthologues appear to have evolved such that only *sept9a* expresses longer isoforms possessing a proline-rich region, while both genes express shorter isoforms primarily consisting of a GTP-binding domain. The predicted polypeptides are highly similar to mammalian SEPT9 proteins, suggesting possible overlapping functions. While zebrafish do not appear to express homologues to human *SEPT9* transcripts 5 and 6, they do express two additional variant 7 transcripts from *sept9b*. It is possible that these transcripts are regulated in a manner similar to *SEPT9_v5* and *v6* [18]. Further studies are required to determine if *sept9a* and *sept9b* have overlapping functions in zebrafish.

RT-PCR analysis of human tissues has shown that a majority of *SEPT9* transcripts are expressed in almost every tissue type tested [6], [7] and cultured cell lines express different combinations of SEPT9 proteins depending on the line [11], [12], [14], [20]. However, whether different SEPT9 polypeptides interact with one another and the individual function of each transcript remains to be determined. We found that zebrafish express multiple *sept9* transcripts from two different genes, and confirmed the role of *sept9* during zebrafish development. Moreover, we observed the same spectrum of phenotypes when we eliminated the largest *sept9a* isoform, *sept9a_tv1*, as when we eliminated all *sept9a* isoforms, providing the first evidence that the smaller isoforms cannot compensate for a lack of the largest isoform.

Recently, a number of *sept9a* transcripts were identified in an analysis of hematopoietic genes isolated from zebrafish kidney marrow [21] and expression of *sept9b* was shown to be increased in embryos overexpressing *etsrp*, a transcription factor required for vasculogenesis and primitive myelopoiesis in zebrafish [22]. This studies support the hypothesis that *sept9* genes play a role in hematopoiesis. However, the pericardial edema, loss of blood circulation and tail malformations observed in both *sept9a* morphant and OE embryos are also consistent with defects in osmoregulation observed when epidermal barrier function is lost [23] or if fish are exposed to toxins that impair homeostasis of the skin or kidney [24], [25]. Zebrafish will be a good model system for future studies examining the roles of various *sept9* isoforms in developmental processes such as hematopoiesis and epidermal development.
